# Temporal epidemiology of microfilaraemia among migrant workers entering Kuwait

**DOI:** 10.1186/1756-0500-1-8

**Published:** 2008-03-19

**Authors:** Saeed Akhtar, Hameed GHH Mohammad, Edwin Michael

**Affiliations:** 1 Department of Community Medicine and Behavioural Sciences, Faculty of Medicine, Kuwait University, PO Box 24923, Safat 13110, Kuwait; 2 Ports and Borders Health Division, Ministry of Health, PO box 32830, Rumaithiya 25410, Kuwait; 3 Department of Infectious Disease Epidemiology, Imperial College Medical School, Norfolk Place, London W2 1PG, UK

## Abstract

**Background:**

There is paucity of published data on the microfilarial infection among migrants from endemic countries entering Kuwait. The primary objectives of this study were to use routine health surveillance data to i) to estimate the prevalence of microfilarial infection in migrant workers to Kuwait and ii) to determine the occurrence of any time trends in the proportions of microfilaria positives among these workers over the recent past.

**Methods:**

Monthly aggregates of microfilaria thick slide test results obtained from routine health examinations of migrant workers conducted at the Ports and Border Health Division of Ministry of Health, Kuwait between January 1, 1992 and December 31, 2006, were available for trend analysis of these time series data.

**Results:**

During the study period, the prevalence (per 100,000) of microfilaraemia positive migrant workers was 48 (1169/2449360). A third-order polynomial regression model of monthly proportions of microfilaraemic workers revealed a significant initial increase (${\hat{\beta }}_{1}$ = 2.976 (± 0.157); *P *< 0.001), followed by a significant declining trend (${\hat{\beta }}_{2}$ = -0.0358 (± 0.002); *P *< 0.001) and a slight but significant upward trend (${\hat{\beta }}_{3}$ = 0.0001 (± < 0.001); *P *< 0.001) towards the end of study period.

**Conclusion:**

This study showed a recent steady but apparently asymptotic decline in the prevalence of microfilarial infection in migrant workers from filarial endemic countries to Kuwait. This may reflect either changes in the socio-economic backgrounds of recent migrants or the effects of recently initiated mass drug administration programs carried out in the endemic countries of origin.

## Background

Lymphatic filariasis caused by the mosquito-borne nematodes, *Wuchereria bancrofti*, *Brugia malayi *and *Brugia timori*, is a major public health problem in many tropical and subtropical regions [1,2]. Of these three nematodes, *W. bancrofti *accounts for more than 90% of this disease burden [1-3]. It inflicts a considerable social and economic burden on many countries of Asia, Africa, the western Pacific and the Americas. The total at-risk population is estimated to be 1307 million in 83 lymphatic filariasis-endemic countries and territories: 65% in the WHO South-East Asia Region and 30% in the WHO African Region. The remaining 5% of microfilaria infected population is distributed among the 3 other WHO regions except the WHO European Region [4]. More than 40 million people have overt clinical disease (mostly hydrocoele, lymphoedema elephantiasis) [1,5]. In addition, even more common than overt abnormalities is the hidden internal damage to kidneys and lymphatic system caused by the worms [6].

Apart from a report on a series of four cases [7], no indigenous case of filarial infection has been reported in Kuwait thus far. However, the presence of the mosquito species, Anopheles and Culex – the vectors of *W. bancrofti*, and a large number of potentially infected migrant workers in Kuwait have raised fears for the possible establishment and local transmission in the country [8]. Despite this, little is presently known regarding either the vectorial capacities of the local mosquito species or the extent of filarial infection in the migrant work force. Here, we take advantage of the routine screening of migrant workers for selected infections, including microfilarial infection, upon arrival in Kuwait from microfilarial endemic regions, to address the second question above, viz. the first large-scale quantification of the filarial infection status of this work population. Specifically, the cumulated data on microfilaria thick slide test results of these workers over the past fifteen years gave us an opportunity in this study not only to undertake 1) the estimation of the prevalence of microfilarial infection in this migrants' population, but also to 2) ascertain if any significant time trend or changes had occurred in the prevalence of microfilarial infection in this population during the recent past.

## Methods

### Study population and data source

Monthly aggregates of microfilaria thick slide test results for migrants entered into Kuwait between January 1, 1992 and December 31, 2006 were available for this study. These migrant workers came from India (31%), Bangladesh (14%), Sri-Lankan (14%), Egypt (12%), Indonesia (9%), Philippine (5%), and Pakistan (5%), while another 10% originated from other countries, including from African counties, such as Tanzania, Mali, Gambia, Sudan [8,9]. For estimation of microfilaraemia prevalence, blood samples were obtained under routine consensual medical examination procedures conducted by the Ports & Borders Health Division of the Ministry of Health, Kuwait, on these workers upon their arrival in the country. For the diagnosis of microfilarial infection, finger-prick blood sample from each worker was taken during daytime, thick blood film made, stained in 10% Giemsa stain for 10 minutes, dried and examined under light microscopy to detect the presence of microfilaria. Each worker was classified either as microfilaraemia negative or microfilaraemia positive regardless of the density and species of microfilaria involved [10].

### Data analysis

The monthly aggregates of daily number of migrant workers tested and daily numbers of microfilaraemia positive were used to generate the monthly series of proportions of microfilaraemic (per 100,000) workers over a period of 180 months from January 1, 1992 to December 31, 2006. We employed standard time series methods to assess and model the presence of statistically significant long term trends in the data. Trend estimation or detection was done by first de-seasonalizing the series using the 13-point (months) moving average filter. Predictive modeling of the trend was then performed following the removal of seasonal effects by initially fitting a locally weighted (Lowess) scatterplot smoother (with bandwidth 0.3) to explore the form of the long-term trend in the relationship between time (months) and monthly proportions of microfilaraemia positive cases [11]. Examination of the results from this exercise suggested the existence of a possible curvilinear temporal trend, and therefore a polynomial regression model was fitted to the deseasonalized data to model the observed monthly proportions of microfilaraemia positive cases with respect to "time", a quadratic term of time (i.e. time^2^) and a cubic term of time (i.e. time^3^)". The goodness-of-fit of the final model was evaluated via residual analysis by plotting residuals against fitted values and also versus the time variable [12].

## Results

**Figure 1:**
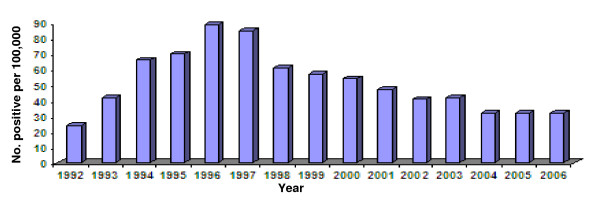
Yearly distribution of proportions of microfilaraemia positive  (per 100,000) migrant workers to Kuwait: 1992–2006.

**Figure 2:**
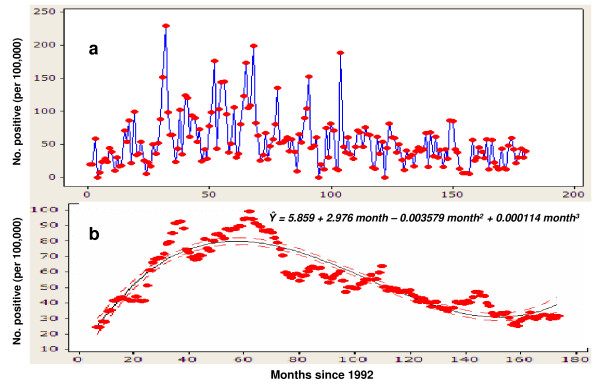
(a) Distribution of proportions (raw data) of microfilaraemia positive migrant workers to Kuwait: 1992–2006. (b) Fit of the polynomial regression model to de-seasonalized data on microfilaraemia positive migrant workers to Kuwait.

During the study period of 180 months from January 1, 1992 to December 31, 2006, 2449360 migrant workers from microfilaria-endemic countries were included in this analysis. Overall prevalence (per 100,000) of microfilaraemia positive migrant workers was 48 (1169/2449360). Total yearly microfilaria infected (per 100,000) cases increased from 24 (95% CI: 18–32) in 1992 to its peak of 89 (95% CI: 74 – 108) in 1996 before it showed a consistent decline to 32 (95% CI: 25–41) in 2004 following which it apparently leveled off till the end of the study period in 2006 (Figure 1). A plot of the actual monthly data, and illustrates both this long-term trend as well as an apparent annual cycle in the numbers of positive microfilaraemia cases detected monthly during the whole of the study period (Figure 2a).

The fit of the Lowess scatter plot smoother (with bandwidth 0.3) to the de-seasonalized monthly data on proportions of microfilaraemia positive cases clearly showed the occurrence of a long-term curvilinear temporal trend in the data with an initial rise in the proportion of cases to a peak by month 60 in 1996 followed by a steady decline thereafter. The corresponding fit of a third-order polynomial regression model with time as the single predictor to these data is portrayed in Figure 2b. Both the overall fit of this model (*F*-statistic = 264.4, *P *< 0.001) (Table 1), and individual terms in the model were statistically significant (*P *< 0.001) with point estimate of each term (± standard error) being ${\hat{\beta }}_{0}$ = 5.859 (± 3.423), ${\hat{\beta }}_{1}$ = 2.9760 (± 0.157), ${\hat{\beta }}_{2}$ = -0.0358 (± 0.002), ${\hat{\beta }}_{3}$ = 0.0001 (± < 0.001). Overall, this model explained 82.9% variation in the monthly proportions of microfilaraemia positive migrant workers (coefficient of determination: *R*^2 ^= 0.829). Thus, the monthly series of proportions of microfilaraemia positive migrant workers revealed a significant (*P *< 0.00 1) initial increase and followed by a significant (*P *< 0.00 1) declining trend, and a slight but apparently significant upward trend towards the end of the 180 months of the study period (Figure 2b). The predicted monthly proportions of filariasis positive cases ranged from 8 to 71 with mean (± SD) of 55 ± 17.

**Table 1:** Polynomial regression model of the deseasonalized monthly proportions (per 100,000) of microfilaraemia positive migrant workers to Kuwait, 1992–2006.

Model terms	Un-standardized partial regression coefficients		t-statistic	*p-value*
			
	Estimate	SE		
Time (${\hat{\beta }}_{1}$)	2.9760	0.157	7.233	< 0.001
Time^2 ^(${\hat{\beta }}_{2}$)	-0.0358	0.002	18.089	< 0.001
Time^3 ^(${\hat{\beta }}_{3}$)	0.0001	< 0.001	15.800	< 0.001
Constant (${\hat{\beta }}_{0}$)	5.859	3.423		

Coefficient of determination (*R*^2^) = 0.829

*F*-statistic for overall significance of the model = 264.4; *P *< 0.001

## Discussion

To our knowledge, this study constitutes one of the largest ever investigations conducted anywhere in the world for estimating and assessing the role of filariasis burdens in migrant workers as a potential source of infection to uninfected areas. This topic is not only of particular relevance to countries, such as Kuwait and the other Middle Eastern countries, which attract a large number of migrant workers from endemic areas. It is also of increasing significance to national filariasis control programmes in endemic counties, wherein mass treatment of regions normally is carried out progressively with cross-boundary migration of people expected to affect intervention effectiveness [13]. We found that the overall prevalence (per 100,000) of microfilaraemic migrant workers was only 48 (1169/2449360) or 0.048% during the entire 15-year study period, a level of infection which may be under the threshold required to establish and sustain parasite transmission [1]. Nonetheless, this result clearly underlines the potential of migrants to serve as sources of new infection in uninfected areas such as Kuwait.

The longitudinal data series based on a 15 year period of observations also uniquely allowed an investigation of the temporal epidemiology of microfilarial infection in workers migrating to the country. This has also specifically enabled us to establish that the filarial risk from migrant workers to the country has reduced dramatically over the past decade, such that the microfilarial infection prevalence in the cohort of workers recruited in 2006 was only around 0.013% (32/230334) in contrast to a peak of 0.069% observed in counterparts in 1996 (Table 1). These results, combined with the uncertainty surrounding the vectorial capacities of the local mosquito species, not only highlight that migrant workers may pose a very small risk for the establishment of filarial transmission in Kuwait presently, but also underscore the value of routine health screening of workers as an effective tool to quantify and track changes in this risk over time.

Although featuring a recent steady decline, our analysis of long-term trends in the data using time series methods revealed the occurrence of an interesting curvilinear pattern in the prevalence of microfilarial infection in migrants over the 15-year study period. Proportions of microfilaraemia positive workers showed an initial increase between 1992 and 1996 and a subsequent steady decline to the present time. The observed initial upward trend in the proportions of microfilaraemia positive workers in this study appears to corroborate previous findings of an increased prevalence of microfilarial infection during the same period in Indian migrant workers in Saudi Arabia [14]. As pointed out in that study, this initial rise during the early to mid nineties may be a function of several factors, including high lymphatic filariasis incidence coupled with un-awareness of the magnitude of this problem and/or absence of any planned control programs in resource-constrained endemic countries of origin of these workers. Following this period, the proportions of microfilaraemia positive migrant workers declined consistently until 2004, and seemed to be leveling off/slightly increasing thereafter. This intriguing consistent decline in the proportions of microfilaraemia positive migrant workers over this decade could reflect the initiation of mass control efforts in the respective endemic countries of origin of these workers. If found to be true, this suggests that sustained mass drug administrations in affected areas over several years could by reducing transmission in those areas also contribute significantly to minimizing the risk of exporting filarial infection into Kuwait and perhaps to other countries in the region. Alternatively, this decline may simply indicate that more workers from a different socio-economic background with lower infection risk compared to that of earlier workers are being enlisted in Kuwait during past one decade or so. We do not have sufficient data at present to investigate this likely change in population characteristics and any potential variations in the prevalence of microfilaraemia between and within the countries of their origins. Nonetheless, whatever the reason for this decline, the slight but significant increase in the microfilaraemia positive proportions of migrant workers towards the end of the time series clearly indicates the need for maintaining the current health screening of these workers to ensure that incidence of infection is not on the increase again. In particular, the need for surveillance is indicated if this increase reflects the outcome of a shift in the health priority of public authorities in the endemic countries resulting in the slow down of filariasis control efforts.

Some limitations of this study need to be taken into account while interpreting the results. First, as only few variables of interest were available for longitudinal analysis, we are unable to evaluate the roles of potential host and environmental factors (such as age, gender, temperature, humidity, rainfall, type and abundance of mosquitoes vectors in different countries) in influencing the observed changes in the prevalence of microfilaraemia. Second, the non-availability of information on exact locations of microfilaraemia positive workers within their countries of origin precluded any spatial or location-based analysis in this study. Furthermore, we did not have access to the data on uniquely identified migrant's country of origin and respective microfilaraemia status of the migrant, thus we could not examine the inter-country variations in prevalence of microfilaraemia. Third, the thick film examination, which is known to have low sensitivity [8], was used for diagnosis of microfilarial infection; it is likely that the true proportion of microfilaraemia positive migrant workers may have been underestimated in this study. Furthermore, filarial infection is known to have diurnal periodicity, with high microfilarial concentration in blood during night [8], and due to administrative constraints, the blood samples from migrants could only be taken during daytime, which additionally might have contributed in the underestimation of true prevalence of microfilarial infection [8,15]. Therefore, the magnitude of the microfilaraemia prevalence reported in this study needs to be generalized to the countries of origin of these workers with caution. This result also suggests that public health authorities in Kuwait may need to consider shifting to more recently introduced immunochromatographic test for circulating microfilarial antigen that has comparable sensitivity and specificity with that of enzyme-linked immunosorbant assay and also does not depend upon nocturnal blood samples [16,17].

## Competing interests

The authors declare that they have no competing interests.

## Authors' contributions

SA conceived, design, analyzed and interpreted data and wrote the first draft of the manuscript. HGHM supervised data collection and reviewed the manuscript. EM guided in analysis and contributed in write up. All the authors have read and approved the final manuscript.
